# Intervention to enhance skilled arm and hand movements after stroke: A feasibility study using a new virtual reality system

**DOI:** 10.1186/1743-0003-4-21

**Published:** 2007-06-23

**Authors:** Jill Campbell Stewart, Shih-Ching Yeh, Younbo Jung, Hyunjin Yoon, Maureen Whitford, Shu-Ya Chen, Lei Li, Margaret McLaughlin, Albert Rizzo, Carolee J Winstein

**Affiliations:** 1 Division of Biokinesiology and Physical Therapy at the School of Dentistry, University of Southern California, Los Angeles, CA, USA; 2 Department of Computer Science, University of Southern California, Los Angeles, CA, USA; 3 Annenburg School for Communication and Integrated Media Systems Center, University of Southern California, Los Angeles, CA, USA; 4 Institute for Creative Technologies, University of Southern California, Los Angeles, CA, USA; 5 Department of Neurology, Keck School of Medicine, University of Southern California, Los Angeles, CA, USA

## Abstract

**Background:**

Rehabilitation programs designed to develop skill in upper extremity (UE) function after stroke require progressive practice that engage and challenge the learner. Virtual realty (VR) provides a unique environment where the presentation of stimuli can be controlled systematically for optimal challenge by adapting task difficulty as performance improves. We describe four VR tasks that were developed and tested to improve arm and hand movement skills for individuals with hemiparesis.

**Methods:**

Two participants with chronic post-stroke paresis and different levels of motor severity attended 12 training sessions lasting 1 to 2 hours each over a 3-week period. Behavior measures and questionnaires were administered pre-, mid-, and post-training.

**Results:**

Both participants improved VR task performance across sessions. The less impaired participant averaged more time on task, practiced a greater number of blocks per session, and progressed at a faster rate over sessions than the more impaired participant. Impairment level did not change but both participants improved functional ability after training. The less impaired participant increased the number of blocks moved on the Box & Blocks test while the more impaired participant achieved 4 more items on the Functional Test of the Hemiparetic UE.

**Conclusion:**

Two participants with differing motor severity were able to engage in VR based practice and improve performance over 12 training sessions. We were able to successfully provide individualized, progressive practice based on each participant's level of movement ability and rate of performance improvement.

## Background

Neurorehabilitation after stroke may include interventions designed to improve functional upper extremity (UE) skills through task-related practice. While amount of practice is an important variable for motor learning [1], variations in direction, timing and speed are needed to optimize the development of skill [2]. Virtual reality (VR) is a promising modality for the creation of favorable practice environments for neurorehabilitation [3-8].

The purpose of this pilot trial was to determine the feasibility of providing individualized, progressive practice of skilled UE arm and hand movements after stroke using VR based tasks. We developed 4 tasks that allowed control of multiple parameters for the purpose of promoting motor skill learning by varying movement direction and speed. We investigated the feasibility of implementing an intervention tailored to each individual's level of movement ability and rate of progression over 12 practice sessions. Preliminary results are reported for two participants with different motor severity.

## Methods

### Participants

Six individuals with hemiparesis were recruited; two with different motor severity were selected for case presentation. Potential participants were screened for inclusion: 1) stroke at least 1 month prior; 2) more than 18 years of age; 3) Mini-Mental Status Exam score ≥ 24; 4) no significant range of motion limitations in the hemiparetic UE; and 5) voluntary movement control to perform the VR tasks. Table 1 includes demographic details for Subjects 102 (severe impairment) and 103 (moderate impairment).

**Table 1:** Participant Demographic Information

Subject ID	Level of Motor Severity	Age (years)	Sex	Time Since Stroke (months)	Type of Stroke	Side of Lesion/Paretic Limb	Hand Dominance Prior to Stroke
102	Severe	88	F	29	Infarct	Right/Left	Right
103	Moderate	73	M	30	Infarct	Right/Left	Right

### Virtual Reality System and Environment

**Figure 1:**
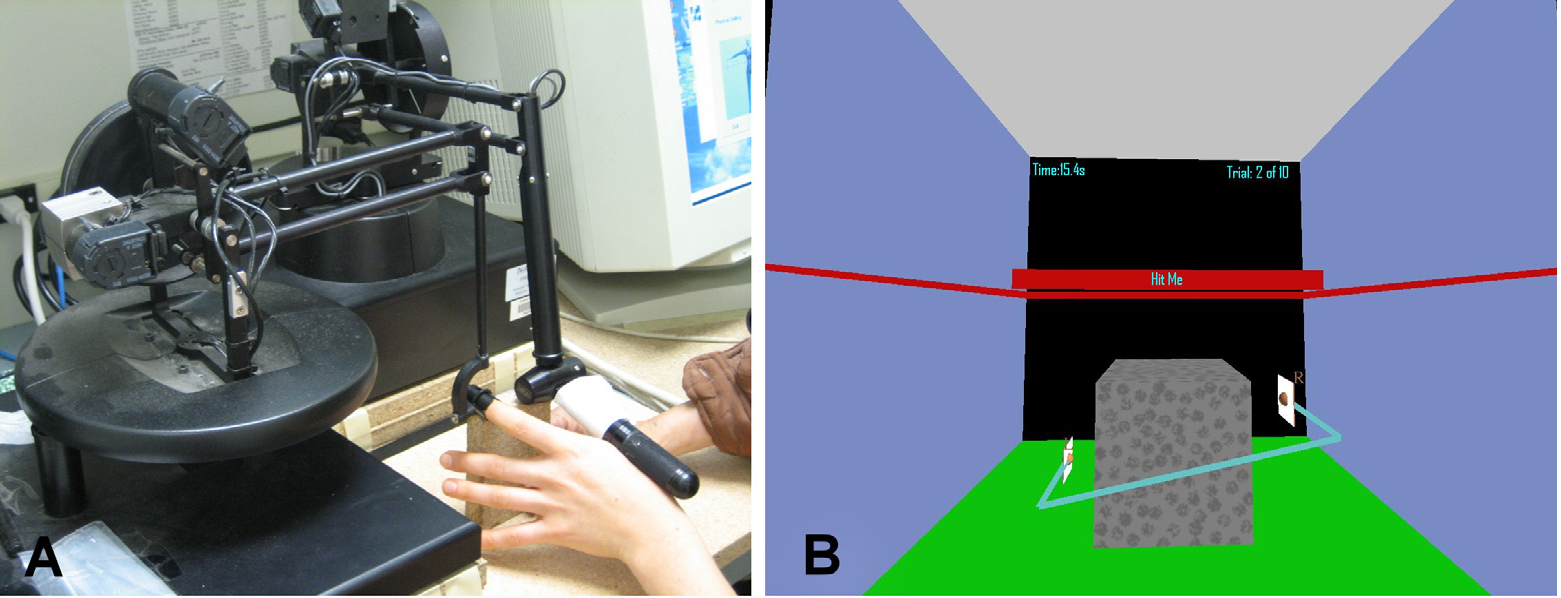
**'Pinch' Task**. A) View of starting position for 'Pinch' including PHANToM device configuration used to calibrate the coordinate system in the virtual environment. Index finger and thumb were held 7 cm apart and parallel to the table. B) View of 'Pinch' scene. Initially, the task required the subject to pick up a cube and place it into a window on the back wall of an enclosed room. Due to technical difficulties, the task was modified. In the new version, the participant picks the object up from the floor, lifts it to a specified height, and places it back on the floor with control. Haptic feedback is provided to both fingers via the PHANToM devices such that the participant has the sense of lifting a real object with mass. There were 10 trials per block; each trial was configured using 8 parameters: cube width (20–40 mm); cube height (20–40 mm); cube length (20–40 mm); mass (50–150 g); dynamic friction (0.5–1.0); static friction (0.5–1.0); stiffness (0.5–1.0); and lift height (20–80 mm). A maximum of 30 seconds was allowed for each trial.

All tasks were displayed using a desktop personal computer and shutter glasses (StereoGraphics) to provide a three-dimensional view of stimuli. To interact with the VR environment in three of the tasks, a 6 degree-of-freedom (DOF) magnetic tracker (Flock of Birds, Ascension Technology) was attached to the participant's hand or to a held object. The fourth task, 'Pinch', was performed using two PHANToM devices (SensAble Technologies) reconfigured to work together. PHANToM 1 was a Premium 1.5/3 DOF model fit with a thimble gimbal replacing the stylus and attached to the end of the index finger. PHANToM 2 was a 6 DOF model with the stylus placed in the web space of the hand and secured to the thumb with an elastic band (Figure 1A). VR tasks were programmed using C++ with Open GL and Ghost libraries.

**Figure 2:**
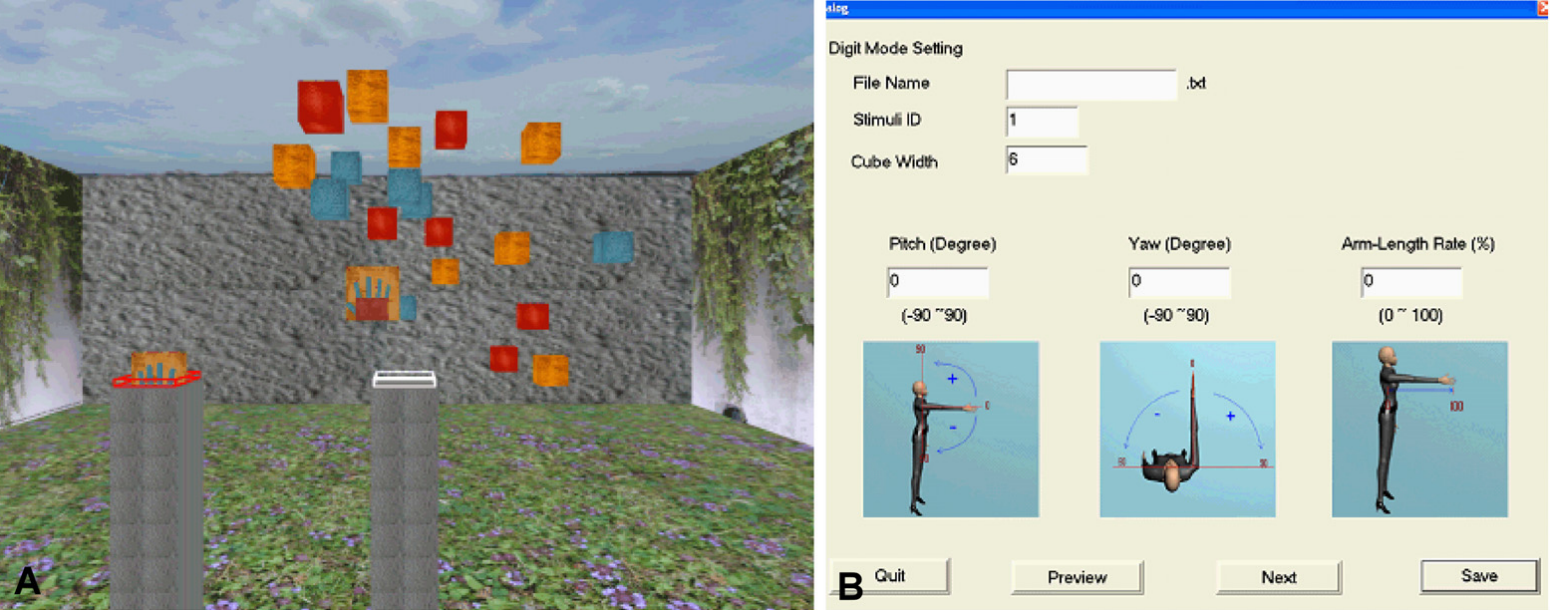
**'Reaching' Task**. A) View of 'Reaching' scene. Each practice block contains 20 cubes (1 cube = 1 trial) presented in relation to each participant's shoulder position. A "virtual hand" corresponds to the location and movement of the paretic hand via a magnetic marker placed either in the palmar surface of a glove or directly onto the dorsum of the hand at the 3^rd ^metacarpal head. Both visual and auditory feedback indicates successful collision of the "virtual hand" with a cube. B) Interface for practice trial configuration. Pitch angle, yaw angle, and percentage of arm length (distance from the acromion to the radial styloid with the elbow extended) were chosen for each cube within a practice block. Practice blocks were designed to address reaching ability using arm lengths ranging from 10% to 120%. A similar interface was used to develop 'Ball Shooting' practice blocks.

**Figure 3:**
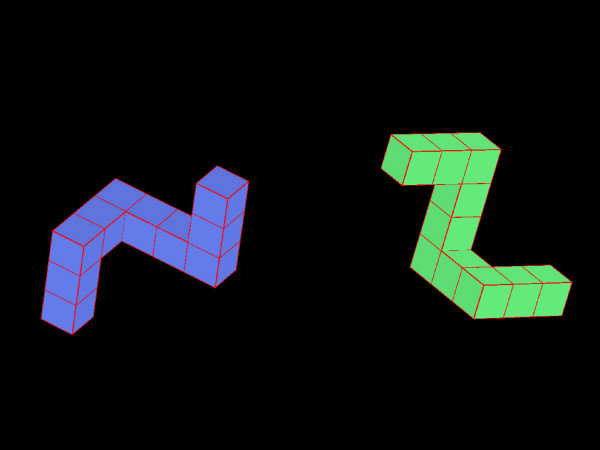
**'Rotation' Task**. The virtual environment consists of two cube configurations that are identical in composition but different in orientation. The participant rotates and laterally moves the green cubes to superimpose them onto the static blue cubes by matching their orientation. Movement of the green cubes is controlled by a magnetic marker attached to a cylinder held in the paretic hand or directly onto the dorsum of the hand at the 3^rd ^metacarpal head. Blocks were configured to require progressively greater amounts of supination ranging from 15° to 150° (from a start position of full pronation). Each practice block contained 20 trials, 10 requiring supination and 10 requiring pronation. A maximum of 60 seconds was allowed for each trial.

Four VR 'games' developed at the University of Southern California Integrated Media Systems Center were adapted to address specific motor deficits common after stroke and to provide a challenging and engaging practice environment. **'Reaching' **requires the participant to reach for static cubes and 'hit' one cube at a time in a participant-selected order (Figure 2A). **'Ball Shooting' **requires the participant to reach and intercept a ball shot from a wall. Both of these tasks were mapped to the individual by presenting stimuli in relation to his/her shoulder location (Figure 2B). **'Rotation' **[9-11] enables forearm pronation and supination movements (Figure 3). **'Pinch' **enables a precision grasp between the thumb and index finger and requires the participant to pick up a cube (Figure 1B). Summary feedback was provided to the participant after the completion of each practice block (10 to 20 trials) in the form of trial success rate and total time.

### Outcome Measures

Behavioral assessments were administered pre-, mid-, and post-training. Severity of motor deficit was determined with the UE portion of the Fugl-Meyer (FM) [12], an impairment-based measure. Functional ability was evaluated with the Functional Test of the Hemiparetic UE (FTHUE) [13] where the individual completes progressively more difficult functional tasks and the Box and Block test (B&B) [14] which requires one to grasp and move 2.5 cm blocks over a 10.8 cm tall barrier. The Stroke Impact Scale (SIS) was administered pre- and post-training to assess participation and health status [15].

### Procedure

Each participant attended 12 training sessions lasting 1–2 hrs/day over 3 weeks. A physical or occupational therapist was present during each session to run diagnostic tests and chose practice blocks and task parameters with the goal to maintain a moderate level of difficulty. If necessary, the therapist provided assistance for task completion, protected joint structures, and/or promoted movement quality.

## Results

### VR Task Performance

Both participants completed all 12 VR practice sessions. Subject 102 (more impaired) was unable to perform 'Pinch' and required physical guidance to complete the other three tasks. Subject 103 (less impaired) practiced all four tasks independently with only occasional assistance. Subject 103 had 18.5% more total training time (7.95 vs. 6.48 hours) and averaged more time on task (39.76 ± 9.38 vs. 32.40 ± 9.3 minutes) and performed a greater number of practice blocks (16.17 ± 4.71 vs. 4.67 ± 1.50 blocks) per training session than did Subject 102.

Subject 103 practiced 'Reaching' blocks targeting 30% to 120% of arm length while Subject 102 practiced blocks ranging from 30% to 50% of arm length. We compared performance on two blocks over practice (Table 2). While the participant with less motor impairment completed the blocks in less time at both time points, both participants reduced block completion time with practice. In 'Ball Shooting', both participants performed blocks that ranged from 10% to 100% of arm length and averaged a greater than 75% success rate at intercepting the ball. Initial diagnostic test results prescribed similar starting ball speed for both participants (0.745 and 0.861 m/s). Practice difficulty was systematically progressed based on individual performance allowing Subject 103 to practice at higher ball speeds (0.745 – 7.011 m/s) over training sessions than Subject 102 (0.861 – 1.650 m/s).

**Table 2:** Time in Seconds to Complete 'Reaching' Blocks Early and Late in Practice

	30% Arm Length			50% Arm Length		
Subject ID	Early	Late	% Change	Early	Late	% Change
102	749.15 (Day 2)	306.71 (Day 12)	-59.1	697.57 (Day 2)	402.50 (Day 6)	-42.3
103	118.29 (Day 1)	41.39 (Day 9)	-65.0	127.82 (Day 4)	46.34 (Day 9)	-63.7

For 'Rotation', both participants began practice on Day 1 with blocks targeting 45° of supination based on diagnostic results. By Day 12, Subject 103 performed blocks targeting a larger supination range (90°) while Subject 102 continued with practice targeting 45°. Finally, Subject 103 was able to practice 'Pinch' while Subject 102 could not. Subject 103 practiced grasping and lifting cubes of various sizes (20 & 40 mm) and weights (50, 100, & 150 g) to the maximal lift height (80 mm).

### Outcome Measures

Physical practice in the virtual environment generalized to different behavioral changes for the two participants (Table 3). Subject 103 showed no change in impairment score (UE FM) but did show functional improvements in grasp and release (B&B, 20% improvement). FTHUE score was unchanged likely due to the ceiling effect at pre-test. Subject 102 did not change impairment level (UE FM) or functional grasp and release (B&B). However, Subject 102 demonstrated a 30% improvement on the FTHUE by completing 4 additional tasks after training. Subject 102 reported less difficulty with arm and hand use after training as measured by the Hand Domain of the SIS, while Subject 103 reported no change.

**Table 3:** Summary of Behavioral Measures

	UE FM Motor Score (66 max)			FTHUE Score* (18 max)			Box & Block** (Mean # Blocks)			SIS Hand Domain (100 max)	
Subject ID	Pre	Mid	Post	Pre	Mid	Post	Pre	Mid	Post	Pre	Post
102	21	22	22	8	8	12	1	0	2	5	35
103	41	43	43	18	17	18	32	37	40	50	50

UE FM: Upper Extremity Fugl-Meyer; FTHUE: Functional Test of the Hemiparetic Upper Extremity; SIS: Stroke Impact Scale

*FTHUE score represents the number of tasks completed.

**B&B value represents the mean of 3 1-minute attempts.

## Discussion

In this report, we describe a newly developed VR system designed to promote UE movement skill in individuals recovering from hemiparesis. Two participants with differing motor severity were able to engage in VR based practice and improve performance over 12 training sessions. We were able to successfully tailor and progress practice content and task difficulty based on each participant's level of movement ability and rate of performance improvement. The feedback provided by the system was useful to the supervising therapist in setting goals, monitoring change in performance, grading task difficulty, and demonstrating performance change to the participant.

Others have reported improvement in UE movement capability in individuals recovering from stroke after training in a virtual environment. Merians et al. [6,16] found improvements in hand function following 2 to 3 weeks of training on VR tasks. The tasks used in those studies focused primarily on hand and finger ability. Our system includes only one task that addresses hand function ('Pinch'), specifically a thumb and index finger pinch, with additional requirements that the grasp be coordinated with a reach movement. Holden et al. [5,17] also demonstrated improved UE function in individuals post-stroke after training reaching movements in a virtual environment. The system used by Holden et al. [5,17] made use of a "virtual teacher" to demonstrate optimal task completion and provide guidance to the user. We did not provide guidance during task performance but provided summary feedback at the completion of each practice block (10 to 20 trials) in order to engage the participant in anticipatory motor planning and problem solving throughout practice.

## Conclusion

The VR system and tasks described in this pilot study provided a challenging practice environment that allowed individually-tailored practice progression. Future work is underway to further validate task design and configuration, develop hypothesis-driven algorithms for optimal task progression, evaluate transfer and persistence of training to real world activities, and incorporate more gaming features.

## Competing interests

The author(s) declare that they have no competing interests.

## Authors' contributions

JCS participated in system design, data analysis and interpretation, and drafted the manuscript. SY, YJ, HJ, and LL participated in system design and data analysis. MW and SC designed and coordinated the experimental protocol and assisted with data collection, analysis, and interpretation. MM and AR conceived of the study and helped in system design, data analysis, and data interpretation. CJW conceived of the study and helped in system design, design of the experimental protocol, data analysis, interpretation, and revision of the manuscript. All authors have read and approved the final manuscript.

## References

[B1] Schmidt RA, Lee TD (2005). Motor control and learning: a behavioral emphasis.

[B2] Gordon JG, Carr J, Shepard R (2000). Assumptions underlying physical therapy intervention: Theoretical and historical perspectives. Movement Science Foundations for Physical Therapy in Rehabilitation.

[B3] Holden MK (2005). Virtual environments for motor rehabilitation: review. Cyberpsychol Behav.

[B4] Weiss PL, Katz N (2004). The potential of virtual reality for rehabilitation. J Rehabil Res Dev.

[B5] Holden M, Todorov E, Callahan J, Bizzi E (1999). Virtual environment training improves motor performance in two patients with stroke: case report. Neurol Rep.

[B6] Merians AS, Jack D, Boian R, Tremaine M, Burdea GC, Adamovich SV, Recce M, Poizner H (2002). Virtual reality-augmented rehabilitation for patients following stroke. Phys Ther.

[B7] You SH, Jang SH, Kim YH, Hallett M, Ahn SH, Kwon YH, Kim JH, Lee MY (2005). Virtual reality-induced cortical reorganization and associated locomotor recovery in chronic stroke. An experimenter-blind randomized study. Stroke.

[B8] Deutsch JE, Latonio J, Burdea G, Boian R (2001). Post-stroke rehabilitation with the Rutgers Ankle System: a case study. Presence.

[B9] Parsons TD, Larson P, Kratz K, Thiebaux M, Bluestein B, Buckwalter JG, Rizzo AA (2004). Sex differences in mental rotation and spatial rotation in a virtual environment. Neuropsychologia.

[B10] McGee JS, van der Zaag C, Rizzo AA, Buckwalter JG, Neumann U, Thiebaux M (2000). Issues for the assessment of visuospatial skills in older adults using virtual environment technology. CyberPsychol Behav.

[B11] van Rooyen AD, Rizzo AA, Buckwalter JG, Larson PJ, Kratz KE, Thiebaux M (2000). The virtual spatial rotation test: a study of psychometric properties. J Int Neuropsychol Soc.

[B12] Fugl-Meyer AR, Jaasko L, Leyman I, Olsson S, Steglind S (1975). The post-stroke hemiplegic patient. 1. A method for evaluation of physical performance. Scand J Rehab Med.

[B13] Wilson DJ, Baker LL, Craddock JA (1984). Functional test for the hemiparetic upper extremity. Am J Occup Ther.

[B14] Desrosiers J, Bravo G, Hebert R, Dutil E, Mercier L (1994). Validation of the Box and Block Test as a measure of dexterity of elderly people: reliability, validity, and norms studies. Arch Phys Med Rehabil.

[B15] Duncan PW, Wallace D, Lai SM, Johnson D, Embretson S, Laster LJ (1999). The stroke impact scale version 2.0. Evaluation of reliability, validity, and sensitivity to change. Stroke.

[B16] Merians AS, Poizner H, Boian R, Burdea G, Adamovich S (2006). Sensorimotor training in a virtual reality environment: does it improve functional recovery poststroke?. Neurorehabil Neural Repair.

[B17] Holden MK, Dyar T, Bizzi E, Schwamm L, Daya-Cimadoro L (2005). Telerehabilitation for motor retraining in patients with stroke. J Neural Phys Ther.

